# Assessing the Efficacy of Restricting Access to Barbecue Charcoal for Suicide Prevention in Taiwan: A Community-Based Intervention Trial

**DOI:** 10.1371/journal.pone.0133809

**Published:** 2015-08-25

**Authors:** Ying-Yeh Chen, Feng Chen, Shu-Sen Chang, Jacky Wong, Paul S F Yip

**Affiliations:** 1 Taipei City Psychiatric Centre, Taipei City Hospital, Taipei, Taiwan; 2 Institute of Public Health and Department of Public Health, National Yang-Ming University, Taipei, Taiwan; 3 Department of Statistics, University of New South Wales, Sydney, Australia; 4 Institute of Health Policy and Management, and Department of Public Health, College of Public Health, National Taiwan University, Taipei, Taiwan; 5 Hong Kong Jockey Club Center for Suicide Research and Prevention, The University of Hong Kong, Hong Kong, SAR China; 6 Department of Social Work and Social Administration, The University of Hong Kong, Hong Kong, SAR China; Medical University of Vienna, AUSTRIA

## Abstract

**Objective:**

Charcoal-burning suicide has recently been spreading to many Asian countries. There have also been several cases involving this new method of suicide in Western countries. Restricting access to suicide means is one of the few suicide-prevention measures that have been supported by empirical evidence. The current study aims to assess the effectiveness of a community intervention program that restricts access to charcoal to prevent suicide in Taiwan.

**Methods and Findings:**

A quasi-experimental design is used to compare method-specific (charcoal-burning suicide, non-charcoal-burning suicide) and overall suicide rates in New Taipei City (the intervention site, with a population of 3.9 million) with two other cities (Taipei City and Kaohsiung City, the control sites, each with 2.7 million residents) before (Jan 1^st^ 2009- April 30^th^ 2012) and after (May 1^st^ 2012-Dec. 31^st^ 2013) the initiation of a charcoal-restriction program on May 1^st^ 2012. The program mandates the removal of barbecue charcoal from open shelves to locked storage in major retail stores in New Taipei City. No such restriction measure was implemented in the two control sites. Generalized linear regression models incorporating secular trends were used to compare the changes in method-specific and overall suicide rates before and after the initiation of the restriction measure. A simulation approach was used to estimate the number of lives saved by the intervention. Compared with the pre-intervention period, the estimated rate reduction of charcoal-burning suicide in New Taipei City was 37% (95% CI: 17%, 50%) after the intervention. Taking secular trends into account, the reduction was 30% (95% CI: 14%, 44%). No compensatory rise in non-charcoal-burning suicide was observed in New Taipei City. No significant reduction in charcoal-burning suicide was observed in the other two control sites. The simulation approach estimated that 91 (95%CI [55, 128]) lives in New Taipei City were saved during the 20 months of the intervention.

**Conclusion:**

Our results demonstrate that the charcoal-restriction program reduced method-specific and overall suicides. This study provides strong empirical evidence that restricting the accessibility of common lethal methods of suicide can effectively reduce suicide rates.

## Introduction

Suicide by charcoal burning reached epidemic levels in Taiwan and Hong Kong in the first decade of the 21st century [1–3]. The first case occurred in Hong Kong in 1998; the method has since spread to Taiwan and several other Asian countries [1, 2, 4]. The most marked rise in charcoal-burning suicide occurred in Taiwan [1], where in less than 4 years, more than 20% of all suicides used charcoal burning (from 69 cases in 1999 to 777 in 2002). In the last 3 years (2011–2013) the method accounted for approximately 25% of suicides in the region [1, 2]. The method is more common in urban areas of Taiwan, where a large number of convenience stores stock barbecue charcoal [5]. Proactive intervention measures to curb the rise of charcoal-burning suicide is a key task of suicide prevention in Taiwan.

Restricting access to suicide means and hotspots is one of the few suicide-prevention measures that have been supported by empirical evidence [6, 7]. Previous studies have assessed the effect of restricting the availability of charcoal on the incidence of charcoal-burning suicides in Hong Kong [8, 9]. In a district of Hong Kong with about 500,000 residents, barbecue charcoal was removed from open shelves and put in locked storage, and customers had to ask a shop assistant to obtain charcoal. Studies found that this purchase barrier (which may result in a 10–15 minute delay) decreased rates of charcoal-burning suicide with no clear evidence of substitution (i.e., adoption of alternative methods). The success of this community intervention study supports the feasibility of reducing charcoal-burning suicide by setting up barriers to its purchase. The purchase barrier may discourage impulsive individuals from continuing to pursue the suicidal act, and it might provide time and opportunities for intervention.

New Taipei City (population: 3.9 million) is the most populated metropolitan city in Taiwan, and in 2010–2012 it had a charcoal-burning suicide rate of about 5.5/100,000 people per year (approximately 216 cases of death by charcoal burning each year). The city initiated a city-wide charcoal restriction program on May 1^st^ 2012 in response to the rising trend of charcoal-burning suicide. The program mandates the removal of charcoal from open shelves to locked storage [10].

Although the pilot study in Hong Kong provided promising evidence that charcoal restriction prevents suicide [8, 9], the scale of the intervention was relatively small. The intervention site had about 500,000 residents, and the control site had approximately the same number in Hong Kong. Each site had only about 20 cases of charcoal-burning suicide recorded during the intervention period [9].

In contrast, our intervention site has a population 8 times that of the Hong Kong study (3.9 million vs. half a million). Therefore, this study has much greater statistical power to test whether this intervention has an effect. The aim of this study was to assess whether removing charcoal from open shelves to locked storage in major retail stores in New Taipei City was associated with a decrease in charcoal-burning suicides and overall suicides.

## Methods

### Ethics statement

The study used only aggregate secondary data that were available openly; no identifiable personal data were used in the study. Ethical approval was thus not required.

### Study design

This is a quasi-experimental study. Two other metropolitan cities—Taipei City (population 2.7 million) and Kaohsiung City (population 2.7 million)—were chosen as control sites to be compared with New Taipei City, the intervention site. New Taipei City, Taipei City, and Kaohsiung City are the three largest metropolitan cities in Taiwan; they are comparable in terms of level of urbanization and access to retail stores. New Taipei City and Taipei City are located in northern Taiwan, and are adjacent to one another. Kaohsiung City is about 400 Kilometers south of Taipei City and New Taipei City.

### Intervention

In Taiwan, the National Suicide Prevention Center coordinates and designs suicide-prevention programs at the national level. Each city also has its own local suicide-prevention programme initiated by the city government to implement city-specific prevention measures. The charcoal-restriction program was initiated by the Department of Health in New Taipei City on May 1^st^ 2012.

With the support of retail stores in New Taipei City, the city required that all charcoal be removed from open shelves. Customers purchasing charcoal have to ask a shop assistant, who would then retrieve charcoal from a locked container. This was designed to make access more difficult for people in a state of heightened distress. No such restriction was implemented in the two control sites.

### Data

We compared the changes in method-specific suicide counts in the intervention period (May 1^st^ 2012- Dec 31st 2013) and pre-intervention period (Jan 1^st^ 2009-April 30th 2012). Official suicide mortality data were extracted from Taiwan’s national cause-of-death file for the study period. Taiwan passed the Personal Information Protection Act on Sep. 26th 2012; hence, individual death record data in 2013 were not released, and researchers can only obtain aggregate data on a weekly scale.

There is no specific code for charcoal-burning suicide in the International Classification of Diseases (ICD) [11]. We used the ICD-10 code X67 (Intentional self-poisoning by and exposure to other gases and vapours) to extract data for charcoal-burning suicide. This code includes suicides by poisoning using gases other than domestic gas, and it is not restricted to charcoal-burning suicides. Previous studies have shown that more than 90% of deaths in this category were charcoal-burning suicides in Taiwan [12]. For simplicity, we refer to these deaths as ‘charcoal-burning suicides’.

Suicides by all other methods were identified using ICD-10 codes X60-X84 (excluding X67) (X60-X84: Intentional self-harm). Previous studies indicated that many deaths categorized as odes X60-X84 (excludin(ICD-10 Codes: Y10-Y34) are likely to be misclassified suicides [5], including charcoal-burning suicides [13]. Therefore, we included such deaths in the analysis. We used the following codes to identify method-specific suicides: i) ICD-10 X67 and Y17 (Poisoning by and exposure to other gases and vapours with undetermined intent) for deaths by charcoal burning, and ii) ICD-10 X60-84 and Y10-Y34, excluding X67 and Y17, for suicides by other methods. We obtained population data for different cities from the Department of Household Registration [14], and we assumed it remained the same in each calendar year. Dataset can be found in the supplement (S1 File).

### Analytic strategy

We first presented method-specific suicide counts and annualized suicide rates for the pre- and post-intervention periods. We then used a nonparametric intensity estimation method to estimate suicide intensity rates before and after the intervention over the study period [15–17]. We modelled the number of cases of a certain method in a certain city until time *t* with a counting process *N*(*t*), with the intensity process *λ*(*t*) ≔ lim*_δt→_*_0+_ E[d*N*(*t*)|*ℱ_t−_*]/*δt* given by λ(*t*) = *P*(*t*)α(*t*), where ℱ_*t*−_ denotes the information available right before time, *P*(*t*) denotes the size of the population at risk at time *t*, and α(*t*) denote the time-dependent location and method-specific suicide intensity/rate function. If the charcoal restriction is effective, then we should be able to see changed patterns in the intensity rate function α at the time when the intervention was implemented. To compute the intensity estimates, we used the R package lpint [18]. To formally test the intervention effect, we used a Poisson generalized linear model (GLM) [19]. In order to account for seasonality, the model includes dummy variables for days of the week and months of the year, which account for any weekday or month effects. The models also include a B-spline function of time (in number of days since the beginning of the study) to account for any general secular time trend in suicide intensity over the study period. For 2013, the dummy variables for weekday were defined as the average of their values over the 7 days. Specifically, the model assumes that the number of charcoal burning or non-charcoal-burning suicide follows a Poisson distribution with a mean depending on the associated covariate variables in the following form,

$$\begin{array}{lll} E\left[y_{i}\right] & = & Pop_{i}T_{i}\text{exp}\{B\left(t_{i}\right)\beta_{tr}+NTP_{i}\beta_{1}^{c}+TP_{i}\beta_{2}^{c}\\ & + & Tue_{i}\;\beta_{1}^{w}+\cdots +Sun_{i}\beta_{6}^{w}\\ & + & Feb_{i}\beta_{1}^{m}+\cdots +Dec_{i}\beta_{11}^{m}\\ & + & postInt_{i}\beta_{1}^{intv}+NTP_{i}postInt_{i}\beta_{2}^{intv}\\ & + & TP_{i}postInt_{i}\beta_{3}^{intv}\;\}. \end{array}$$

In the above expression, *Pop*_*i*_ denotes the population size, *T*_*i*_ the length of the observation period, *B(t*_*i*_) the row vector containing the values of the B-spline basis functions evaluated at the center of the observation period *t*_*i*_, *NTP*_*i*_ and *TP*_*i*_ the two dummy variables for the New Taipei City and Taipei City respectively, *Tue*_*i*_,…,*Sun*_*i*_ the 6 dummy variables for the variable weekday, *Feb*_*i*_,…,*Dec*_*i*_, the 11 dummy variables for the variable month, *postInt*_*i*_ the indicator of intervention. The parameters *β* represent the regression coefficients. In particular, $\beta_{1}^{intv}$ represents the potential intervention effect in the reference city Kaohsiung, $\beta_{2}^{intv}$ represents the extra intervention effect in the New Taipei city compared to Kaohsiung, and $\beta_{3}^{intv}$ represents the extra intervention effect in the Taipei city compared to Kaohsiung. The order of the spline function for secular trend was fixed at 4, which corresponds to the commonly used cubic splines. The knots of the splines were evenly spaced, with the number of knots selected using the Akaike Information Criteria (AIC) [20].

After fitting the Poisson GLM, we use a dispersion test [21] to check for potential overdispersion of the data relative the Poisson model, if overdispersion was detected, we would use the negative binomial GLM to refit the data. After fitting the Poisson or negative binomial GLM model, we also check the probability integral transform (PIT) residuals [22] for goodness of fit of the model to the data. If the fit is sufficient, the PIT residuals are expected to be uniformly distributed, which can be checked graphically using e.g. the histogram plot, or formally using e.g. a chi-squared test. If the fit were insufficient then we would include more explanatory variables such as the interactions of the variables to the model.

The Poisson and negative binomial GLMs can be estimated using the maximum likelihood approach. For practical computations, we used the R programming language [23]. To fit the Poisson GLM, we used the glm function; to test for over-dispersion after fitting a Poisson GLM, we used the dispersion test function from the AER package [21]. To fit the negative binomial GLM, we used the glm.nb function from the MASS package [24].

To estimate the number of lives saved by the intervention, we used a simulation based approach. Specifically, using the estimated model for the charcoal-burning suicide data in New Taipei City, we simulated the charcoal-burning suicide counts that would have been observed over the intervention period if the intervention were not implemented, and then we calculated the difference between the simulated total number of cases and actual total number of cases observed. The difference was one simulated value of the number of lives that could have been saved. The simulation was repeated *B* = 10,000 times. The median of the simulated values was taken as an estimate of the number of lives saved, and the 2.5th and 97.5th percentiles of the simulated values as the lower and upper limits of a 95% CI for the number of lives saved, respectively.

## Results

Table 1 provides the numbers and rates of method-specific suicide before and after the intervention. In the intervention site, suicide by charcoal burning decreased from an average annual rate of 6.2 per 100,000 of population in the pre-intervention period to 3.9 per 100,000 after the measure, a decrease of 37% (95% CI: 17%, 50%). The change in non-charcoal-burning suicide was not prominent (from 12.3 to 11.9 per 100,000, a decrease by 3%) in New Taipei City (Table 1). This resulted in a decrease in the overall suicide rate in New Taipei City from 18.6 to 15.8 per 100,000.

**Table 1:** Number and rate of suicides during the pre-intervention (Jan 1^st^ 2009- April 30^th^ 2012) and post-intervention period (May 1^st^ 2012-Dec. 31^st^ 2013) in New Taipei City, Taipei City and Kaohsiung City.

Suicide methods	Period	Intervention site		Control sites			
		New Taipei City		Taipei City		Kaohsiung City	
		N	Rate	N	Rate	N	Rate
Charcoal burning	Pre-intervention	808	6.2	305	3.5	490	5.3
	Post-Intervention	256	3.9	111	2.5	219	4.7
Non-charcoal burning	Pre-intervention	1598	12.3	945	10.8	1381	14.9
	Post-Intervention	783	11.9	471	10.6	684	14.8
All methods	Pre-intervention	2406	18.6	1250	14.3	1871	20.2
	Post-Intervention	1039	15.8	582	13.1	903	19.5

Among the control sites, Taipei City’s charcoal-burning suicide rate decreased from 3.5 to 2.5 and Kaohsiung City’s decreased from 5.3 to 4.7, but the extent of decrease was not as prominent as in New Taipei City. There was not much change in the overall suicide rates in the two control sites: 14.3 to 13.1 in Taipei City and 20.2 to 19.5 in Kaohsiung City.

**Fig 1:**
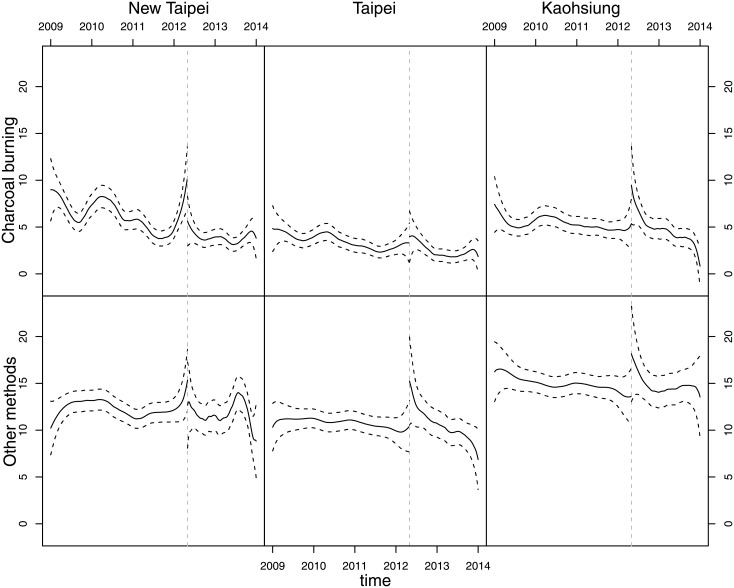
Estimated method-specific suicide intensity (smoothed suicide rates) before (Jan 1^st^ 2009- April 30^th^ 2012) and after May 1^st^ 2012 (May 1^st^ 2012-Dec. 31^st^ 2013) in New Taipei City, Taipei City and Kaohsiung City respectively.

Fig 1 gives non-parametric estimates of intensity of charcoal-burning suicide and non-charcoal-burning suicide before and after the implementation of the restriction measure. Only New Taipei City showed a significant reduction in the charcoal-burning suicide intensity at the time of implementation. Fig 1 shows that the drop in charcoal-burning suicide rate in New Taipei City was statistically significant; the 95% CI for the suicide intensity immediately after the intervention date does not include the estimated suicide intensity immediately before the intervention date, indicating that there was a downward change point on the suicide intensity curve at the start of the intervention program. However, such a downward change point was not found on the suicide intensity curves in the control cities. Charcoal-burning suicide in New Taipei City remained at a relatively low level until the end of 2013, when there was a slight rebound.

**Fig 2:**
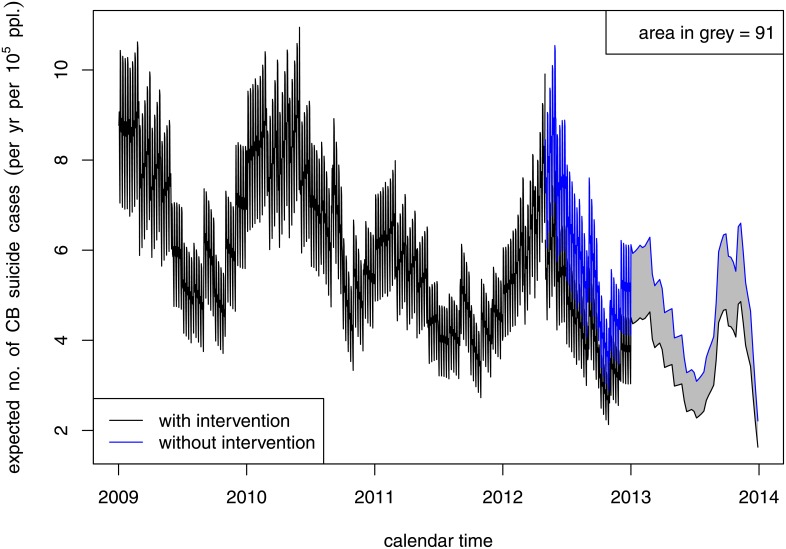
Projected and actual suicide rate trend for charcoal burning suicide in New Taipei City.

Table 2 shows the results of fitting the time-series models incorporating seasonality and secular trends using pre-intervention charcoal burning suicide rate in Kaohsiung city as the reference group. By the best fitting Poisson GLM for charcoal burning suicides, there was a statistically significant decrease in the rate of charcoal-burning suicide after the intervention in New Taipei City compared with Kaohsiung City. The extent of the decrease for charcoal-burning suicide in New Taipei City relative to Kaohsiung city was estimated to be 30% (1-exp(-0.36) = 30%), 95% CI [14%, 44%], P = 0.001). The decrease in charcoal-burning suicide in the intervention site was not accompanied by a compensatory rise in non-charcoal-burning suicides in New Taipei City. The overall decrease in the suicide rate of New Taipei City after the intervention was estimated to be 12% (1-exp(-0.13) = 12%), 95% CI [2%, 21%]. The simulation approach estimated that 91 (95%CI [55, 128]) lives in New Taipei City were saved during the 20 months of the intervention (Fig 2).

**Table 2:** Relative changes in method specific suicide rates after the intervention, estimated by the time series regression models incorporating secular trends in New Taipei City, Taipei City and Kaohsiung City.

Method of suicide	City			
		Estimates	S.E.	P value
Charcoal burning	New Taipei City	-0.36	0.11	0.001***
	Taipei City	-0.23	0.14	0.10
	Kaohsiung City ^c^	0.05	0.27	0.85
Non-charcoal burning	New Taipei City	-0.03	0.06	0.68
	Taipei City	-0.01	0.07	0.85
	Kaohsiung City	-0.21	0.18	0.25
All methods	New Taipei City	-0.13	0.06	0.02*
	Taipei City	-0.06	0.06	0.39
	Kaohsiung City	-0.16	0.15	0.30

Note:

*P <.05;

** P <.01;

*** p <.001

Note: pre-intervention charcoal burning suicide rate in Kaohsiung City was treated as the reference

**Fig 3:**
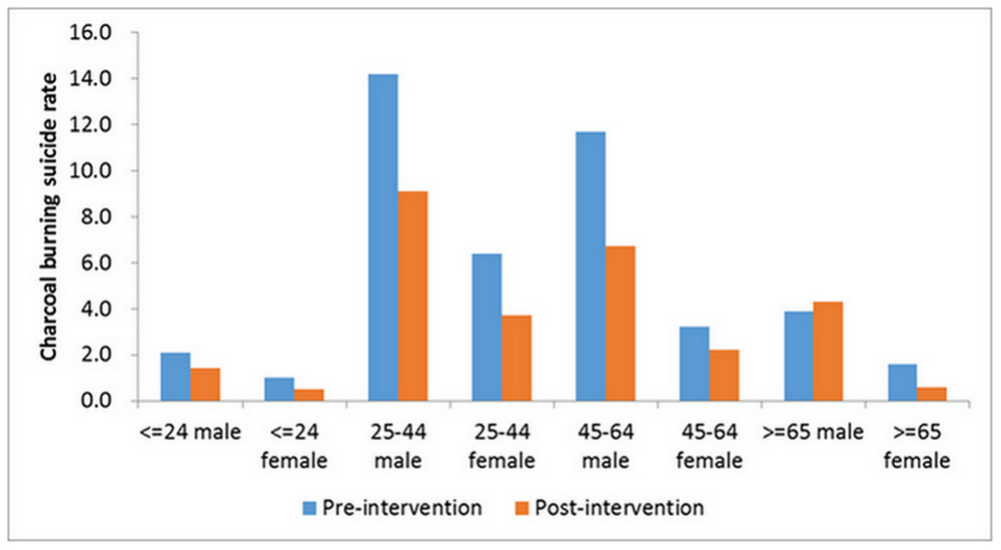
Changes in rates of charcoal burning suicide in different socio-demographic groups in New Taipei City before and after the charcoal restriction program.

Fig 3 illustrated the changes in charcoal burning suicide rates before and after the restriction measure in different age and sex groups. Except for men older than 65 years old, a general decrease in charcoal burning suicide rates was found in all age and sex groups. The most marked decrease was found in men aged 25–64 and female aged 25–44.

## Discussion

### Main findings

Our analysis finds empirical evidence that the charcoal-restriction program reduced the number of suicides in New Taipei City. Suicide by charcoal burning was on the rise before the inception of the program. After the program was started, the rate of charcoal-burning suicide decreased significantly. Moreover, the decrease in charcoal-burning suicides in New Taipei City did not result in any substitution effect (i.e., switching to other methods of suicide when charcoal became difficult to access). Hence, after the intervention, there was a decrease in not only the rate of charcoal-burning suicides, but also the overall suicide rate in New Taipei City. Rates of charcoal-burning suicides in Taipei City and Kaohsiung City, the control sites, did not decrease during the study period.

It was estimated that about 91 lives were saved as a result of the charcoal-restriction program during the intervention period. Our finding supports the effectiveness of this charcoal-restriction program in curbing suicide.

### Strength and limitations

Very few suicide-prevention programs have been empirically validated [6, 7, 25, 26]. One important reason for the scarcity of evidence-based suicide-prevention measures is that suicide is a rare event. Thus, the intervention has to have very large scale to obtain meaningful results [27]. This large-scale community-based charcoal-restriction program provides evidence that method restriction helps prevent suicide. Suicide by charcoal burning has been a rising public health issue in many Asian countries [1, 2]. These findings provide crucial information and experience to curb the continuing trends of this suicide epidemic.

However, our results need to be interpreted in light of the following limitations. First, as described in the method section, we were not able to differentiate charcoal-burning suicides from deaths using other sources of non-domestic gas, such as car exhaust. However, charcoal-burning suicide accounted for more than 90% of all suicides from non-domestic gas poisoning in Taiwan [13], and there was no report of a prominent rise in gas suicide using methods other than charcoal burning during the study period.

Second, other unmeasured social factors may have contributed to the observed efficacy of the program. However, any social factors (such as celebrity suicide or economic recession) that were associated with suicide rates should have affected the control sites as well. The fact that both Taipei City and Kaohsiung City did not witness a significant decrease in charcoal-burning suicide points to the validity of the current observation. Having said that, it is still possible that certain region-specific factors may have only affected the intervention site and not the control sites.

Third, the intervention was not compulsory, and many independently owned convenience stores and small businesses did not comply with the measure. Given budget and manpower limitations, only chain supermarkets were regularly audited for compliance with the restriction policy. Moreover, it is possible that rather than purchasing charcoal from convenience stores; suicidal cases might use charcoal stocked at home. Given very limited and expensive living space in metropolitan cities in Taiwan, it is quite uncommon for Taiwanese to stock charcoal at home. However, any kind of ‘non-compliance’ would drive our results towards not finding an effect. Thus, if non-compliance was a factor, the actual effect would be even stronger than our estimate.

Fourth, New Taipei City and Taipei City are adjacent cities; those who were determined to die by charcoal burning suicide in New Taipei City can easily get charcoal from convenience stores in Taipei City. The awareness of the New Taipei City restriction program by Taipei City residents may have changed their behaviours due to competition effect. However, if this did happen, the net effect would drive the observed results towards the null. Indeed, we observed a decrease in charcoal burning suicide in Taipei City as well, even though no restriction program took place there.

Lastly, the measurement of the intervention effect relies on ecological analysis. We were not able to determine if the decrease in charcoal burning suicide in the intervention site was actually due to charcoal restriction (i.e. ecological fallacy). However, this limitation is inherent to all types of community based intervention studies when the intervention elements are not applied to each individual but to the population as a whole.

### Interpretation and implications

Our findings provide strong empirical evidence to indicate the efficacy of reducing charcoal-burning suicides through restricting access to charcoal. This program did not completely eliminate suicide means (i.e., charcoal) but made the purchase of charcoal more time consuming to creating a barrier. Customers would be forced to make contact with shop attendants and would have to wait for their assistance in order to get a pack of charcoal. These barriers may have precluded potential victims especially the impulsive ones from purchasing charcoal; they may have deferred their decision or switched to a less-lethal method (such as medication overdose).

To implement method restriction, it is crucial to assess whether there is a popular and readily available competing method and whether the fatality of the alternative method is lower than that of the restricted suicide method [6, 28]. In New Taipei City, the top three most common methods of suicide were charcoal burning (31.6%), hanging (26.3%), and jumping (17.1%) during 2010–2012. Because the lethality of hanging and jumping is higher than suicide by charcoal burning [6, 29], the possibility of method substitution should be seriously considered and closely monitored. In our analysis, there was no increase in any other methods of suicide during the intervention period, indicating no or little evidence of method substitution.

It is likely that suicidal individuals may have a preference for a particular method [28, 30]. This sort of method preference may be particularly true for charcoal-burning suicide. Portrayed as a peaceful and painless way of dying, suicide by charcoal burning has attracted a new cohort of individuals who might not have died without this method [4, 31, 32]. This resonates with one previous interview study that showed more than half of charcoal-burning suicide attempters had not considered alternative methods [33]. The desirability of a particular method of suicide may be an important contributing factor in the success of this charcoal-restriction program.

Our results are consistent with a previous intervention trial in Hong Kong [9]. That study found a reduction of approximately 50% in suicide by charcoal burning in the intervention site. Although the results were promising, the intervention has not been extended to whole Hong Kong because chain store management is reluctant to proceed with the measure; they are concerned about lack of space and the loss of business from the measure.

The process of initiating restriction measures in Hong Kong was different from New Taipei City. In Hong Kong, shop clerks and residents in the intervention site were not aware of the trial. The Hong Kong program was known to the research team and the managers of the chain stores only. In New Taipei City, the measure was initiated by the City Government. The media was sceptical of the measure; hence it was widely publicized and had received some criticism before the implementation. Whether the publicity of this large-scale community-based intervention program has any positive or negative impact on the program outcome is yet to be known. However, it is possible that the extensive publicity of this measure may discourage people from using this method or prevent them entering any retail store to get a bag of charcoal because they are worried about the extra hurdle.

In addition to charcoal removal, the New Taipei City Government used retail stores as points to raise public awareness of suicide and mental disorders. Brochures and pamphlets containing psycho-education and psychiatric care resources were distributed to supermarkets. Shop clerks were encouraged to keep an eye on the behaviours of customers who purchased charcoal; if the customers looked distressed, they could actively provide them with these brochures. It is possible that the observed effect of charcoal restriction may in fact be the combined effect of many measures conducted during the period.

Indeed, the implementation of this charcoal-restriction program requires a high level of cooperation from retail stores and the community. It is necessary to get the support of supermarket chains and day-to-day implementation by store employees to place the bags of charcoal in locked storage. Community members might not like the inconvenience and might be sceptical of the efficacy of the intervention. People tend to believe that suicide is inevitable; removing access to one method of suicide would make people use other methods.

In fact, charcoal burning suicide decreased in the control sites as well, although to a much smaller extent. It is possible that the diffusion of charcoal burning suicide was saturated after more than ten years of spreading in the community; hence, with or without intervention, it was more likely to observe a decrease rather than an increase in rate. However, the prominent decrease in the intervention site was statistically significant even when the overall downward secular trend was incorporated in the model. One other possible factor that may contribute to the decrease of charcoal burning suicide in control sites could be the increased awareness and strengthened prevention efforts in control sites due to widespread publicity of the charcoal restriction program in New Taipei City.

Subgroup analysis shows that the most marked decrease in charcoal burning suicide was found in males aged 25–64 and females aged 25–44. In other words, the restriction measure is particularly effective for the subgroups that suicide by charcoal burning was very common. This is consistent with previous observation that a precondition for ‘means restriction’ to take effect is that the method to be restricted should be a common method of suicide. It should be noted that the rate of charcoal burning suicide in men older than 65 years of age didn’t decrease after the restriction measure; it is possible that older men tend to have high suicide intention [25], hence the restriction measure may not alter their behaviors.

Hopefully, the encouraging results from this study can stimulate more community support for this program in Taiwan and in other Asian countries troubled by charcoal-burning suicide. Increasing awareness of the suicide problem and the recognition of the importance of community-based intervention will generate a more conducive environment for suicide prevention.

## Supporting Information

S1 File Dataset used in the current analysis. (XLSX)
